# Sigma frequency dependent motor learning in Williams syndrome

**DOI:** 10.1038/s41598-017-12489-y

**Published:** 2017-12-01

**Authors:** Andrea Berencsi, Róbert Bódizs, Ferenc Gombos, Szandra László, Ilona Kovács

**Affiliations:** 1 Laboratory for Psychological Research, Pázmány Péter Catholic University, Mikszáth tér 1, Budapest, H-1088 Hungary; 2 Department of General Psychology, Pázmány Péter Catholic University, Mikszáth tér 1, Budapest, H-1088 Hungary; 3 Department of Medical Psychology, Institute of Behavioural Sciences, Semmelweis University, Nagyvárad tér 4, Budapest, H-1089 Hungary; 4 Institute for Methodology of Special Education and Rehabilitation, Eötvös Loránd University Bárczi Gusztáv Faculty of Special Education, Ecseri út 3, Budapest, H-1097 Hungary; 5 PhD School of Mental Health Sciences, Semmelweis University, Balassa u. 6, Budapest, H-1089 Hungary

## Abstract

There are two basic stages of fine motor learning: performance gain might occur during practice (online learning), and improvement might take place without any further practice (offline learning). Offline learning, also called consolidation, has a sleep-dependent stage in terms of both speed and accuracy of the learned movement. Sleep spindle or sigma band characteristics affect motor learning in typically developing individuals. Here we ask whether the earlier found, altered sigma activity in a neurodevelopmental disorder (Williams syndrome, WS) predicts motor learning. TD and WS participants practiced in a sequential finger tapping (FT) task for two days. Although WS participants started out at a lower performance level, TD and WS participants had a comparable amount of online and offline learning in terms of the accuracy of movement. Spectral analysis of WS sleep EEG recordings revealed that motor accuracy improvement is intricately related to WS-specific NREM sleep EEG features in the 8–16 Hz range profiles: higher 11–13.5 Hz z-transformed power is associated with higher offline FT accuracy improvement; and higher oscillatory peak frequencies are associated with lower offline accuracy improvements. These findings indicate a fundamental relationship between sleep spindle (or sigma band) activity and motor learning in WS.

## Introduction

**Figure 1:**
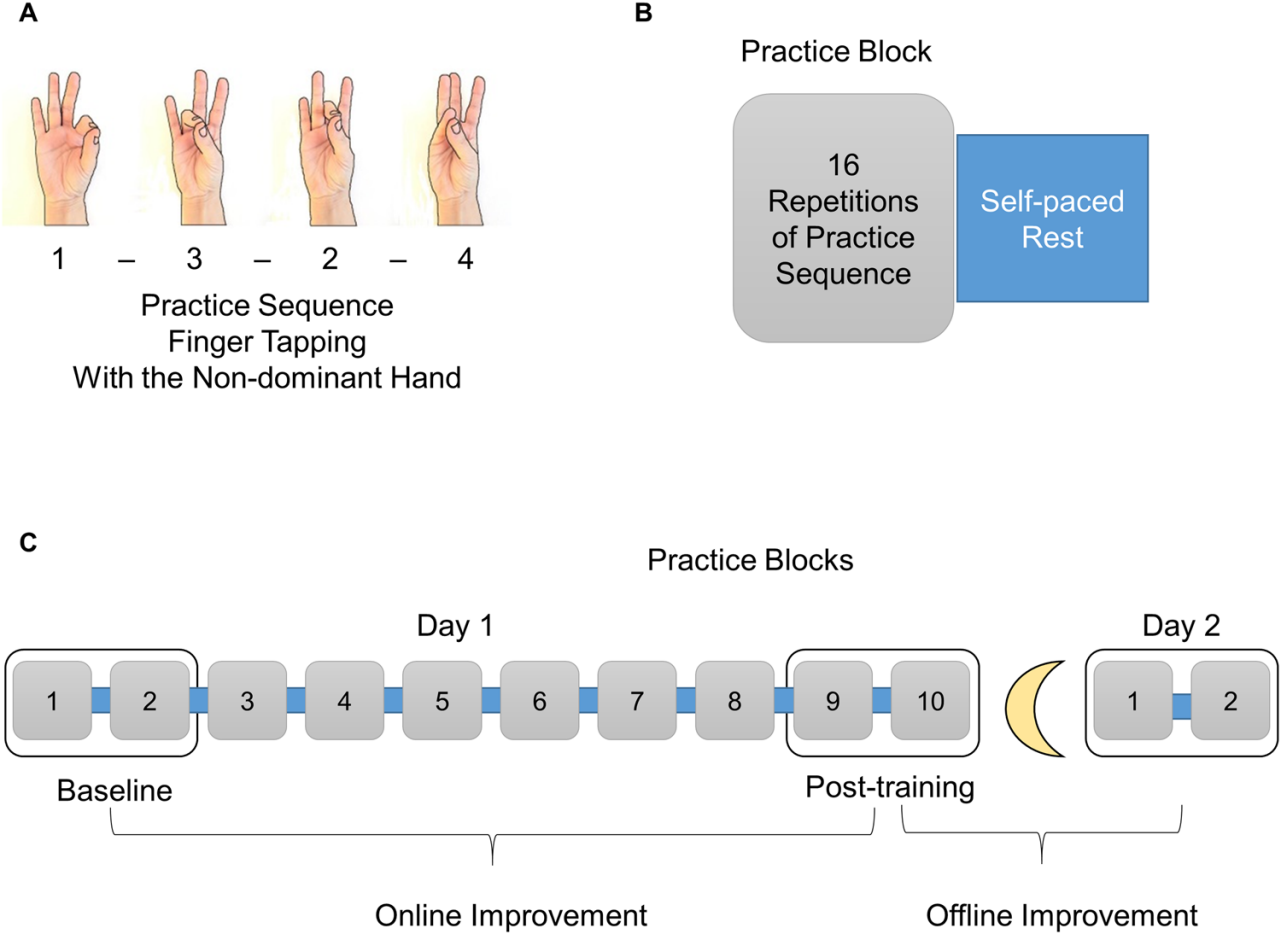
Motor learning task. (**A**) A four-element FT sequence, practiced with the non-dominant hand. Thumb is touched with the index, ring, middle and little fingers in this predetermined order. A “data glove” consisting of metal ring electrodes placed on each fingertip detects the order and timing of taps. (**B**) Each practice block is composed of 16 repetitions of the four-element practice sequence, and followed by a self-paced rest period. Beginning and end of a practice block is signalled by a computer-generated “beep” sound. (**C**) 10 practice blocks are carried out on Day 1. The mean of the first two practice blocks is considered as baseline performance. Online improvement is defined as the difference between the baseline and the mean of the last two practice blocks. Offline improvement is defined as the difference between the mean of the last two blocks on Day 1 and the mean of the first two blocks on Day 2.

Motor learning, particularly explicit learning of fine motor sequences has multiple phases. Within-session (online) gains occur during practice, both in accuracy and in speed. The online phase is followed by a consolidation phase in which motor performance improves offline, without any further practice (Fig. 1). Offline consolidation is brain-state dependent: time spent awake results in retention of performance acquired during practice, while off-line gains are sleep-dependent both in speed and in accuracy in adults^1–3^. Changes in delta, sigma and beta activity in NREM sleep, and in REM characteristics have been found following practice^4,5^. There is a positive correlation between offline gains in the explicit sequential finger tapping (FT) task and sigma band NREM activity in the 13–15 Hz spindle range (in spindle amplitude, number and density)^4,6–12^. Post-sleep improvement in FT is correlated with spontaneous delta and fast-sigma oscillations in the supplementary motor area, contralateral to the trained hand^6,7,13^. Taken together, recent evidence seems to confirm that sleep spindles, especially fast sleep spindles contribute to the activation of the neural network involved in offline consolidation of fine motor sequences^4,7,11,14–16^.

In addition to post training sleep, sleep quality before learning also affects motor learning capacity in healthy individuals^17^. Interestingly, sleep spindle characteristics preceding learning are related to baseline performance and offline improvement in children when learning motor finger-sequences^18^. As a general trait, sleep dependent learning is correlated with EEG activity in the 8–16 Hz band during NREM sleep, which is genetically determined and stable within individuals, and across nights^19,20^.

**Figure 2:**
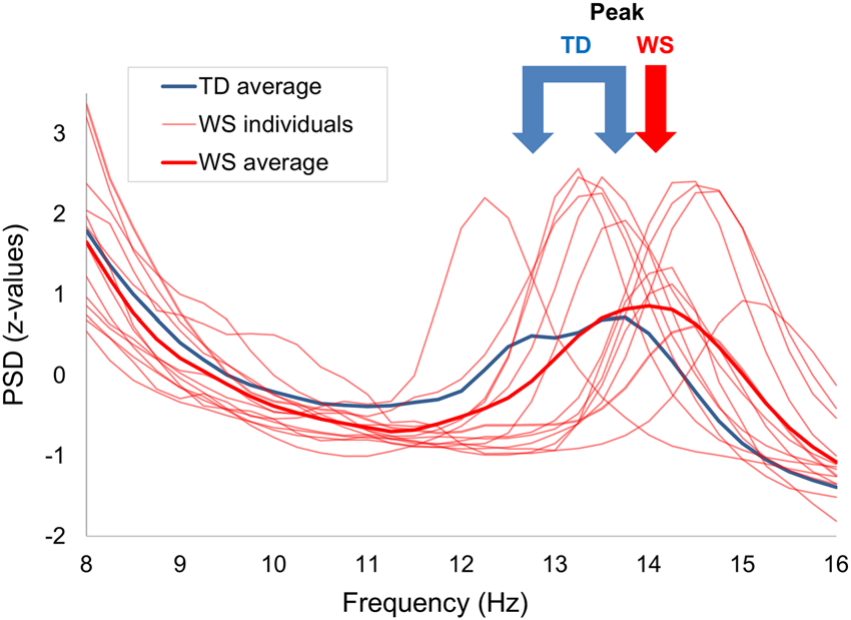
Focus of the present study: the broadband sigma range (8–16 Hz) in NREM sleep. Normalized sigma power as expressed in z-scores of EEG activity in WS subjects and group averages (WS and TD) at derivation Cz. Spectral power densities of artifact-free, Hanning-tapered 4 second EEG epochs were calculated via the Fast Fourier Transformation method and averaged for all-night NREM sleep (data from^15^)/TD data shown as a reference from^21^. The 8–16 Hz range was normalized in a derivation- and individual-specific manner by z-transformation^19^. TD sigma activity typically has two peaks (it is also true for the individual subjects) which could be referred to as the slow and fast sleep spindle peak frequencies, correspondingly. The slow spindle peak is usually missing or greatly reduced in WS patients and generally the second (fast spindle) peak is at a higher frequency in WS than in TD subjects^21^.

As we have shown earlier, the majority of the above mentioned, motor learning related sleep characteristics are altered^21^, and the capacity to improve during long-term FT learning is significantly limited in Williams syndrome (WS)^22^. WS is a genetically determined neurodevelopmental disorder due to a microdeletion on chromosome 7 in the q11.23 region, and it is characterized by mild to moderate intellectual disability, hypersociability, attention deficits, and problems with visuospatial processing^23–25^. Delayed motor development, gross and fine motor deficits throughout the lifespan are common findings in WS^25,26^. With respect to sleep, an atypical sleep pattern, including prolonged sleep latency, sleep maintenance problems and fragmented sleep^27–29^, decreased total sleep time^30,31^, increased slow wave sleep (non-REM stage 3 and 4 sleep)^28,30–32^; decreased REM sleep percentage and reduced cyclicity in the sleep architecture^31^ has been found in WS. We have found WS-related alterations in the broadband sigma (8–16 Hz) NREM sleep EEG spectral profiles: decreases and increases in low (<13 Hz) and high (>13 Hz) sigma power, respectively, as well as increased oscillatory sigma peak frequencies^21^. This pattern has a striking stability in time, suggesting the acceleration of thalamocortical oscillatory dynamics during NREM sleep in WS^21^ (Fig. 2).

Based on the above findings, here we investigate the hypothesis that the reduced capacity to improve in a motor learning task is related to the altered neural activity during sleep in WS. Specifically, we test the relationship between motor learning and 8–16 Hz NREM sleep in WS. We employed a two-day practice version of the sequential FT task in WS and TD groups, and we compared their learning capacities. The same WS individuals participated in whole night ambulatory polysomnographic recordings earlier, and we analysed the relationship between polysomnographic and FT measures. Our main assumption is that the peculiar sleep characteristics of WS (namely, decreased low sigma, increased high sigma, as well as increased sigma peak frequency Fig. 2) will influence learning capacity in terms of both accuracy and speed in a sleep-dependent learning task. More specifically, we predict lower offline motor memory improvement when WS-specific alterations in the NREM sleep EEG sigma spectral profiles are pronounced.

## Results

### Motor learning performance

#### Accuracy

**Figure 3:**
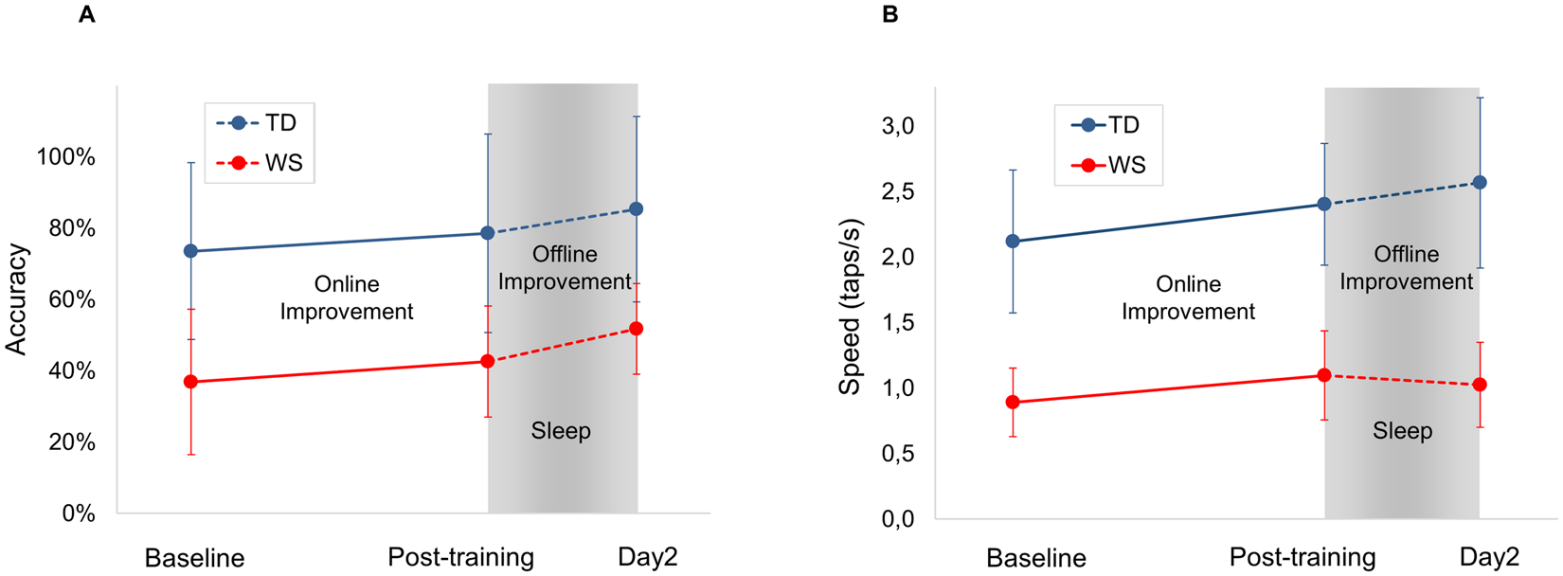
(**A**) Online and offline improvement in accuracy in the sequential FT task. (**B**) Online and offline improvement in speed in the sequential FT task in WS and in TD subjects. Baseline performance is significantly different, while improvements are comparable in WS and TD with respect to accuracy and online improvement in speed. Error bars show standard deviation.

A significant main effect of group (WS < TD: F_(1,30)_ = 23.939; p = 0.00003), a significant main effect for baseline/online/offline improvement (F_(2,60)_ = 48.614; p = 2.8098 × 10^−13^, and a significant group × baseline/online/offline interaction (F_(2,60)_ = 7.465; p = 0.001) was present. Baseline was significantly lower in WS than in TD (p < 0.05), but no significant difference in online and offline improvement was found between WS and TD groups (p > 0.05). Therefore, in terms of accuracy, baseline performance was different, while online and offline improvement in the sequential FT task was comparable in WS and TD (Fig. 3A).

#### Speed

There was a significant main effect for WS and TD groups (F_(1,30)_ = 71.648; p = 1.9077 × 10^−9^), with a significant main effect of baseline/online/offline improvement (F_(2,60)_ = 113.894; p = 3.7359 × 10^−21^) and with a significant group × baseline/online/offline interaction (F_(2,60)_ = 18.605; p = 5.1657 × 10^−7^). Baseline was significantly lower in WS than in TD (p = 0.05), and there was no significant difference in online improvement (p > 0.05). Offline improvement showed a marginal difference between the two groups (p = 0.065) (Fig. 3B).

Lower baseline performance predicted higher online (practice-dependent) gains in terms of both accuracy (r = −0.74, p = 0.001) and speed (r = −0.59, p = 0.015) of motor learning in TD, but not in WS participants. Similar negative correlations between offline and online improvements were detected in both groups (TD r = −0.63, p = 0.01, WS r = −0.67, p = 0.005) with respect to speed, and in TD with respect to accuracy (r = −0.74, p = 0.001).

#### Correlations between sleep parameters and motor learning performance

**Figure 4:**
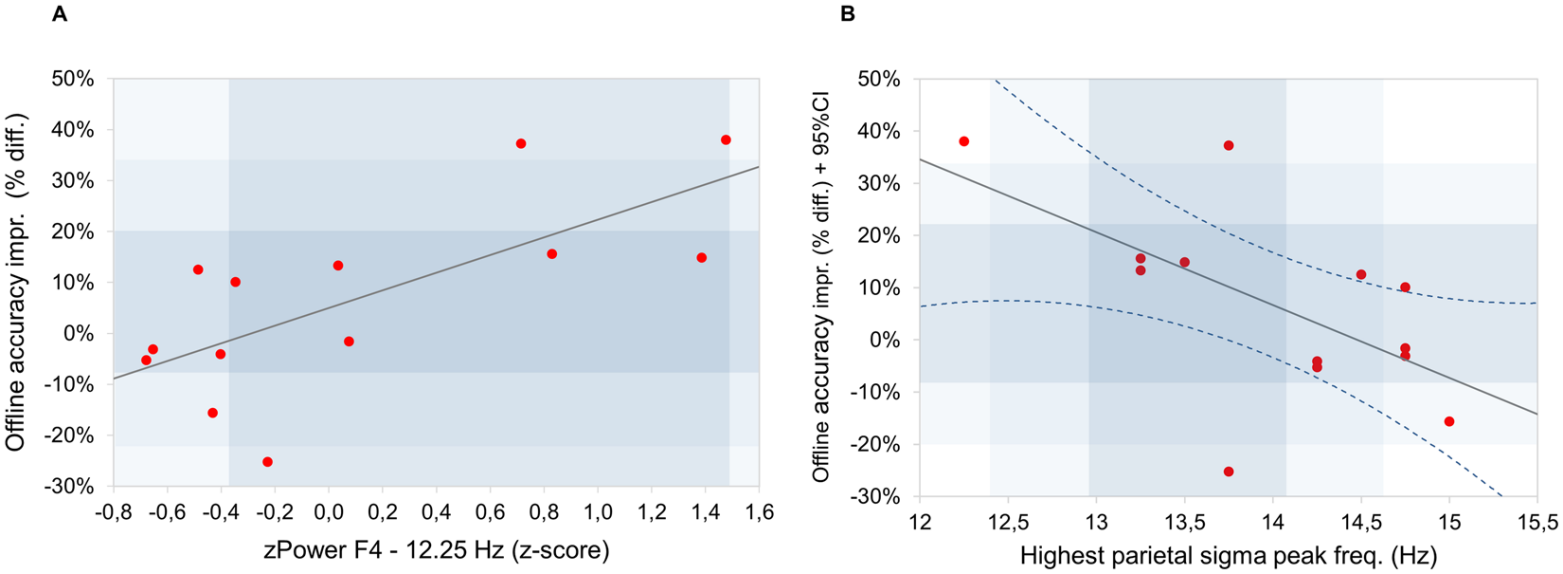
(**A**) Positive correlation between z-scores of 12.25 Hz NREM sleep EEG power, and Day 1 to Day 2 offline motor accuracy improvement in WS participants. The z-score of the 12.25 Hz power is based on the individual-specific normalization of 8–16 Hz spectra^20^ in the right frontal derivation (F4). Offline FT accuracy improvement is expressed in terms of percent change from Day 1 to Day 2. Light and dark grey indicates ±1 and ± 2SD of the corresponding variable in TD participants, respectively. SD of TD spectral data are from ref.^21^. Note that higher 12.25 Hz power is associated with higher offline FT accuracy improvement. (**B**) Negative correlation between parietal sigma peak frequency and Day 1 to Day 2 offline motor accuracy improvement in WS participants. Highest parietal sigma peak frequency is the frequency at which the highest observable local maxima are found in the 8–16 Hz NREM sleep EEG power spectra of WS participants. Offline FT accuracy improvement is expressed in terms of percent change from Day 1 to Day 2. Light and dark grey indicates ±1 and ±2 SD of the corresponding variable in TD participants, respectively. SD of TD spectral data are from ref.^21^. Note that higher oscillatory frequencies are associated with lower offline accuracy improvements.

Correlations between z-scores of the NREM sleep EEG 8–16 Hz spectra and motor learning in WS subjects are reported in terms of significant Rüger’s areas: continuous zones of descriptive (p < 0.05) significances in the frequency (8–16 Hz with 0.25 Hz resolution) × EEG location (10–20 system) matrices for which the area as a whole can be considered as significant (see details in Methods). No significant Rüger’s areas were revealed for baseline motor accuracy and speed performances. Bins between 11 and 13.5 Hz correlated positively with offline accuracy improvement, forming a continuous Rüger’s area involving all EEG derivations. Maximal effect occurred at derivation F4 and the 12.25 Hz frequency bin: r = 0.69, p = 0.004 (see Supplement 1 and Fig. 4A).

Bins in a similar, but somewhat lower frequency range (however still consistent with the low sigma range: 9.75–12.25 Hz) correlated positively with online (practice-dependent) motor speed improvement. The Rüger’s area involved the majority of EEG locations (F3, F4, Fz, C3, C4, Cz, T3, T4, T5, T6, P3, P4, Pz, O1, O2, Oz), except the anterior frontal ones (Fp1, Fp2, Fpz, F7, F8). No significant Rüger’s areas were revealed for baseline motor accuracy and speed performance.

Further characteristic features of the NREM sleep EEG broadband sigma spectral profiles are the oscillatory peak frequencies. Decreased offline accuracy gains in WS participants with higher oscillatory peak frequencies are indicated by the negative correlation between the highest parietal sigma peak frequency and offline motor accuracy improvement in the FT task (r = −0.52, p = 0.03, (Fig. 4B). We found no other significant correlations between sigma peak frequencies and motor learning performances in WS participants.

## Discussion

The purpose of the present work was to contribute relevant evidence on the relationship between sleep and motor learning. As it has been shown in typically developing subjects, sleep spindles contribute to the activation of the neural network involved in offline consolidation of fine motor sequences^4,6–12^. Here we studied a group of subjects whose spindle activity is modulated at the group level (a single and shifted peak in the sigma domain, see Fig. 2), and the peak frequency modulation has a great individual variability (also Fig. 2). These two factors together gave us a unique possibility to test whether spindle modulation results in altered learning performance. Our results seem to attest to that, providing unique evidence for a relevant relationship between spindles and motor learning: sigma activity closer to the TD range is associated with better offline learning in WS.

We tested whether the specific alterations of broadband sigma (8–16 Hz) activity in WS^21^ are associated with deficits in offline fine motor learning. We found that two of these alterations are indeed related to affected offline motor learning phase in the FT task. The previously reported decrease in low sigma range of the z-normalized spectra of WS participants is involved in the efficiency of achieving offline performance gains. WS participants, characterized by a smaller amount of decrease in z-normalized low sigma power show the highest offline gains in motor accuracy (Fig. 4A). The second finding is related to the accelerated sigma peak frequency in WS, which correlates negatively with offline motor accuracy gains. In other words, WS participants, characterized by more accelerated sigma peak frequencies exhibit less effective offline consolidation (Fig. 4B). We also report an unpredicted finding, namely, a positive correlation between the individual values of z-normalized low sigma power and online (instead of offline) gains in motor speed.

In terms of motor learning, WS participants show significantly lower baseline performance as compared to TD participants with respect to both accuracy and speed. On the other hand, low baseline in WS is not associated with sigma band activity as found previously in typically developing children, where, e.g., more slow sleep spindles are associated with lower baseline when polysomnography is administered during a three-day-learning session^18^. Our results indicate that WS and TD participants are not different in terms of online improvement at the group level in speed or accuracy in the initial phase of motor learning. Similarly, intact online learning performance has been found in other studies that focused on populations with sleep alterations. When individuals with obstructive sleep apnoea were compared to healthy controls, there was no difference in improvement during first day practice in a finger sequence task^33^. Moreover, schizophrenia patients were not different from healthy controls in accuracy^34,35^, and subjects with attention-deficit hyperactivity (ADHD) disorder were not different in speed from TD controls^36^ in terms of within-session improvement.

Surprisingly, online improvement in speed is correlated with low sigma power in WS in our present study. Given that most of the studies in the field focus on the relationship between offline gains and changes in post-training sleep, the studies on the interrelation between sleep characteristics and baseline and/or online improvement are scarce. A recent study of King *et al*.^37^ shows that cerebral activation during initial online learning predicts sleep dependent motor performance improvements in the elderly. They hypothesize that a given level of activation in motor networks involving the putamen, cerebellum and parietal cortex is required to induce sleep related consolidation. In healthy adults^38^, performance changes are related to the increased activation of putamen and medial temporal lobe including the hippocampus. During the initial online phase of motor sequence learning, performance improvement as measured by consistency is correlated with the interaction between the striatal and hippocampal systems that in turn predicts offline improvement in typically developing adults. That is, brain activation associated with performance gains during online learning may trigger and, in turn, predict offline performance changes. Since the above studies did not administer polysomnographic recordings, only an indirect relationship may be hypothesized between online improvement and low sigma band activity in the present study. Since brain activation during motor learning seems to be disease specific in spite of similar behavioural characteristics^39^, further studies need to clarify the functional relevance of given brain areas and their polysomnography correlates in motor learning in WS.

With respect to offline improvement, a pattern of dissociation is frequently observed in age related changes or in disrupted sleep: while online performance is retained, offline improvement is compromised^9,33–36,40^. Unlike in these previous studies, offline learning in WS was comparable to that of the TD participants from Day 1 to Day 2 in our study (Fig. 3). Individual differences in offline accuracy gains were correlated with WS-specific NREM sleep EEG sigma activity features (low sigma power and highest peak frequency) (Fig. 4B). These results are consistent with findings in recent study of individuals with ADHD. Participants with ADHD had lower baseline, but similar performance on Day 2 post-test compared to TD. Authors found positive correlation between offline improvement and slow sleep spindle activity (12–13.5 Hz) during the night after training in ADHD, but not in TD controls. Participants with higher relative power in the frequency band related to slow sleep spindles, the frequency band that is in turn the more altered in ADHD, had better overnight improvement^41^. Similarly, in patients with major depression, overnight learning was associated with slow frequency spindle activity (10.5–12.5 Hz), and this relationship was true for healthy control participants too^42^. Furthermore, typically developing children with lower initial performance but more slow spindles and slower slow waves improved more in accuracy overnight^18^.

In our study, we found no association between sigma band activity and offline gain in speed in WS. We analysed speed and accuracy measures separately, since our previous study showed a lower baseline and impaired learning in WS with respect to speed during a five day motor training^22^. Here we analyse an initial phase of learning, and we find only a marginal difference between WS and TD groups in terms of offline improvement in speed, not reaching significance, and not correlated with sigma activity in WS. Other studies allowing for a dissociation between speed and accuracy with respect to online and offline improvement show an incoherent picture. In TD children, improvement in accuracy in a FT task was sleep-dependent, while improvement in speed was not. In conditions with disrupted sleep, e.g., when individuals with ADHD were trained on a sequential FT task, they expressed delayed gains at 24 h and at 2 weeks retention in speed, but did not improve compared to baseline in accuracy^36^. On the other hand, in schizophrenia patients, while sleep dependent learning was compromised both in speed and accuracy^34,35^, offline performance gains were correlated only with the stage 2 NREM sleep length but not with spindle activity when measured in the 4th quarter of the night in schizophrenia patients^35^.

In spite of the exciting findings, our study also has several limitations. The number of available participants is relatively small since WS is a rare disorder. Furthermore, the difficulty of the motor task for young individuals with WS, and the presence of motor symptoms (such as tremor) in adult individuals with WS also put restriction on the number of participants. Consequently, the relatively large age-range in the study is a possible limitation of the results. While the identification of fast spindles in children is controversial^43^ we think that the IAM method used for spindle detection is able to detect fast spindles also in children^44^.It might occur as another limitation that data submitted to correlation analysis were not obtained at the same time point. However, since the studied sleep characteristics are genetically determined and stable within individuals, and across nights, this factor may not affect the interpretation of our data. Another limitation is that are not presenting a direct comparison in terms of sleep and behavioural data in typically developing control participants. However, the aim of the present study is to reveal the relationship between a known and confirmed alteration in sigma activity in NREM sleep and motor learning capacity in WS, and the studied sleep alteration is not present in TD subjects.

Our results are consistent with studies indicating the importance of sigma band activity in NREM sleep in motor memory consolidation, where it has been shown that among individuals with a neurodevelopmental disorder, characteristics of sigma band activity closer to that of TD subjects results in superior learning, with retained/higher slow sigma activity being associated with higher offline improvements^41,42^. We have also found a more general effect by demonstrating that trait-like NREM sigma activity characteristics (not associated with the motor training) may influence motor memory consolidation in addition to changes in post training sleep. Further studies are necessary to explore the more detailed relationship between sigma band activity and online learning, and the dissociation between speed and accuracy during motor learning. Notwithstanding that IQ is not a direct predictor of learning in the motor domain^12^, interpretation of data in relation to IQ may also give new insights. The WS-specific alterations of sigma band activity are of potential interest for those aiming to unravel the neural roots of the individual differences in motor learning performance in participants with WS or other neurodevelopmental disorders.

## Methods

### Ethics statement

The present study was approved by the Social Sciences Ethical Review Board of the Budapest University of Technology and Economics and was conducted according to the approved guidelines. Informed consent was obtained from adult participants and the parents of participating children in the study.

### Participants

Motor learning:16 individuals with WS (6 males, 10 females) participated in the study. The age-range was 11 to 27 years, mean age was 18,4 years (SD:5,5 years). Three participants with WS were left-handed, two were mixed handed, and eleven were right handed as measured by tool use. WS diagnosis of all WS participants was confirmed by fluorescent *in situ* hybridization tests showing deletion on chromosome band 7q11.23. 11 participants took part also in a previous motor learning study^16^. Furthermore, 16 typically developing individuals (6 males, 10 females), age range 11–29 years, mean age 18,4 years (SD:5,6 years) participated in the study. All TD participants were right handed and did not report sleep disruptions. None of the participants were professional musicians or had skeletal deformities that could influence motor performance.

Polysomnography (PSG): 20 participants with WS took part in an earlier polysomnographic study^21^. A subgroup of these WS participants (n = 13) also took part in the motor learning experiment. 4 males and 9 females (age range 11–27 years, mean age: 18,6 years, SD: 5,6 years; 9 right-handed, 3 left-handed, 1 mixed-handed) with WS participated both in the ML and the PSG the studies.

Participants were allowed to go to bed at will, and were not awakened during the PSG recording, and during the night following acquisition. Prior to PSG recording, an adaptation night with the same conditions served the subjects to get used to the experimental settings. Subjects were only included in the FT experiment if a minimum of 6 hours of night sleep was reported previously, and between Day 1 and Day 2 of testing. Sleep duration was assessed by self-reports of the participants. Sleep architecture of WS participants of the present experiment is shown in Table 1.

**Table 1:** Sleep architecture of the Williams syndrome subjects.

WS sleep (N = 13)	Average	SD
Record duration (min)	556,00	65,15
Sleep duration (min)	484,33	52,38
Sleep efficiency (%)	87,50	7,13
Wake duration (min)	71,67	44,39
Relative wake duration (%)	12,50	7,13
WASO (min)	34,26	33,39
Sleep latency (min)	40,08	24,92
NREM duration (min)	379,67	42,94
Relative NREM duration (%)	78,49	4,78
S1 duration (min)	8,05	6,17
Relative S1 duration (%)	1,65	1,20
S2 duration (min)	282,82	41,49
Relative S2 duration (%)	58,34	5,09
SWS duration (min)	88,79	22,61
Relative SWS duration (%)	18,50	4,97
REM duration (min)	104,67	26,43
Relative REM duration (%)	21,51	4,78
REM latency (min)	79,36	41,94
Number of Sleep cycles	4,77	1,36
Average REM period (min)	23,48	9,33
Average sleep cycle (min)	97,03	19,45

SWS, slow wave sleep; WASO, wake time after sleep onset; NREM, non-rapid eye movement; REM, rapid eye movement. Values are group means ± SD.

## Experimental Design

### Motor learning experiment

#### Task and design

The motor learning task was a four elements FT task performed with the non-dominant hand. Participants touched their thumb with the index finger, followed by the ring finger, middle finger and little finger, always in this same sequence (Fig. 1). Data acquisition started after participants performed three successful sequences consecutively. They practiced 10 blocks of 16 repetitions on the first day, and 2 blocks of 16 repetitions on the second day. One of the participants with WS practiced only five blocks on the first day. Participants were allowed to rest between practice blocks at their own pace to avoid fatigue^45^.

#### Data acquisition

Data were collected using a custom-made data glove including metal rings placed on each fingertip. The data glove was connected to a laptop computer where a Java based data acquisition software stored and processed the timing and order of finger taps. Since 11 out of 16 participants took part in a previous long term motor learning study^22^, that database was included in the present study for performing a new analysis focusing on online and offline learning in the fast phase of learning.

#### Dependent variables

We monitored performance in speed and accuracy during acquisition. Speed was defined as the number of finger taps in a second (taps/s). Accuracy (%) was defined as the ratio of the number of finger taps in correctly performed sequences to all sequences. Baseline was calculated as the mean performance of the first two blocks on the first practice day, and post-training performance as the mean performance of the last two blocks on the first practice day. Online (practice-dependent) improvement was calculated as a difference between the mean of the first two blocks of a daily session and the mean of the last two blocks of the same session. Offline improvement was calculated as the difference between the last two blocks of a daily session, and the first two blocks on the following day.

Baseline performance, online and offline improvement were calculated with respect to speed and accuracy.

#### EEG recordings and analyses

A subgroup of WS participants from our sleep EEG study^21^ were enrolled in the FT learning session protocol. We analysed sleep EEG data with high interindividual variability and proven intra-individual stability (the sleep EEG fingerprints) of this subgroup in relation with the FT measures of the current study. Sleep EEG data were derived from whole night ambulatory polysomnographic records as follows. Home sleep was recorded according to the participants preferred sleeping habits by using a 32 channel SD-LTM Hardware together with the BRAIN QUICK System PLUS EVOLUTION software (Micromed, Italy). We recorded EEG according to the 10–20 system (Jaspers, 1958) at 21 recording sites (Fp1, Fp2, Fpz, F3, F4, F7, F8, Fz, C3, C4, Cz, P3, P4, Pz, T3, T4, T5, T6, O1, O2, Oz) referred to the mathematically linked mastoids. We also recorded bipolar EOG, ECG and submental as well as tibialis EMG. Signals were high-pass filtered at 0.15 Hz and low-pass filtered at 250 Hz by 40 dB/decade anti-aliasing hardware input filters. Data were collected with 22 bit resolution and with an analogue to digital conversion rate of 4096 Hz/channel (synchronous). The firmware applied a further 40 dB/decade anti-aliasing digital filter which low-pass filtered the data at 463.3 Hz. The digitized and filtered EEG was subsequently undersampled at 1024 Hz. We also applied a 50 Hz digital notch filter performed by the recording software. Data were exported and converted to EDF^46^ before further analyses.

We visually scored sleep recordings according to standard criteria^47^ in 20 s epochs. Next, we manually removed the 4 s epochs containing artifacts before further automatic analyses. Average power spectral densities were calculated by a Fast Fourier Transformation (FFT) algorithm applied to the 50% overlapping, Hanning-tapered, artifact-free 4 s epochs of whole night stages 2–4 NREM sleep. We used z-scores of 8–16 Hz spectra in the statistical analyses. We introduced z-transformation in order to follow previous approaches in the field^21^, as well as to control for potentially simultaneous differences in general EEG amplitude and delta power, the latter being supported by our own results^31^ and indirectly by the reports of an increased SWS in WS^31,32^. The z-transformation is individual and derivation-specific as described below. Spectral values (µV^2^/0.25 Hz) between 8 and 16 Hz (33 bins of 0.25 Hz each) are averaged (m) and the standard deviation calculated (σ). Then the original frequency bins were replaced by the individual- and derivation-specific z-scores by using the z = (x-m)/σ formula, where x is the actual value of the frequency bin (in µV^2^/0.25 Hz) to be transformed. This normalization results in series with a mean of 0 and standard deviation of 1, varying between 8 and 16 Hz, and reflecting the individual- and derivation-specific shapes of the spectra, previously termed as spectral EEG fingerprints of sleep^19^, characterized by strong genetic determination^14^ and proven reliability (repeatability) in both healthy^19^ and WS participants^21^.

Spectral peaks were processed automatically as follows: the zero-crossing points of the first order derivatives of the z-scores of 8–16 Hz spectra were considered as locations of spectral peaks if the second order derivatives were negative at these frequencies (local maxima in mathematical terms). Spectral peak processing was performed on the averaged z-scores of the left and right frontal (F3 and F4), as well as on the left and right parietal (P3 and P4) derivations. Slow peaks were defined as the slowest peak in the frontal derivations, while fast peaks as the fastest ones in parietal channels.

### Statistics

#### Finger tapping task

We performed two-way mixed ANOVA (group × baseline/online/offline) with repeated measures on baseline/online/offline regarding all dependent variables. Mauchly’s Test of Sphericity indicated that the assumption of sphericity had not been violated, therefore correction was not performed for sphericity of the repeated measures. Post hoc test were performed by using the Fisher LSD method. To investigate the relationship between baseline, online and offline improvement, we calculated correlation coefficients between these measures both in speed and accuracy. A p-value of 0.05 was set as the significance level for all statistical tests.

#### Correlations between spectral values and learning performance

We calculated correlations between spectral values and learning performance as follows. We correlated individual-specific slow and fast spectral peak frequencies, as well as binwise z-scores with FT measures (Pearson product-moment correlation coefficients). The binwise calculations result in an inflation of type I statistical error, thus, we applied the Descriptive Data Analysis procedure^47^ to control this effect. As a first step, continuous regions of descriptive, uncorrected (p < 0.05) significances derived from binwise z-power vs motor performance correlations are defined in the frequency (between 8 and 16 Hz, with 0.25 Hz resolution) × EEG location (according to the 10–20 system) matrices. These regions are termed Rüger’s areas and are further tested in order to make global confirmatory statements with controlled uncertainty (at least one of the null hypotheses in the Rüger’s area is wrong). In order to refuse this global null hypothesis, at least half of the significant correlations in the respective Rüger’s area have to be significant at the level of 0.05/2 (0.025). As our hypotheses were directional (formerly reported WS-specific features of the 8–16 Hz spectra^15^ were hypothesized to negatively influence motor learning), we used one-tailed tests when assessing significances. In addition, we tested the Rüger’s areas for outliers: we visually screened the scatterplots of maximal correlational effects in the potential Rüger’s areas and removed the areas where extreme values caused spurious associations from further analyses.

## Electronic supplementary material

Supplementary Figure S1.
