# Supplementary material for: Sigma frequency dependent motor learning in Williams syndrome
Source: Sci Rep. 2017 Dec 1;7:16759. doi: 10.1038/s41598-017-12489-y (PMC5711805; doi:10.1038/s41598-017-12489-y)
Supplement: Supplementary file 1 — Supplementary Figure S1. [file 41598_2017_12489_MOESM1_ESM.pdf]

Title: Sigma frequency dependent motor learning in Williams syndrome

Authors: Andrea Berencsi[1,4*], Róbert Bódizs[1,2,3], Ferenc Gombos[1,2], Szandra László[1,5], Ilona Kovács[1,2]

Affiliations:

[1] Laboratory for Psychological Research, Pázmány Péter Catholic University, Mikszáth tér 1, Budapest, H-1088, Hungary

[2] Department of General Psychology, Pázmány Péter Catholic University, Mikszáth tér 1, Budapest, H-1088, Hungary

[3] Department of Medical Psychology, Institute of Behavioural Sciences, Semmelweis University, Nagyvárad tér 4, Budapest, H-1089, Hungary

[4] Institute for Methodology of Special Education and Rehabilitation, Eötvös Loránd University Bárczi Gusztáv Faculty of Special Education, Ecseri út 3, Budapest, H-1097, Hungary

[5] PhD School of Mental Health Sciences, Semmelweis University, Balassa u. 6, Budapest, H-1089, Hungary

*Corresponding author: berencsi.andrea@barczi.elte.hu

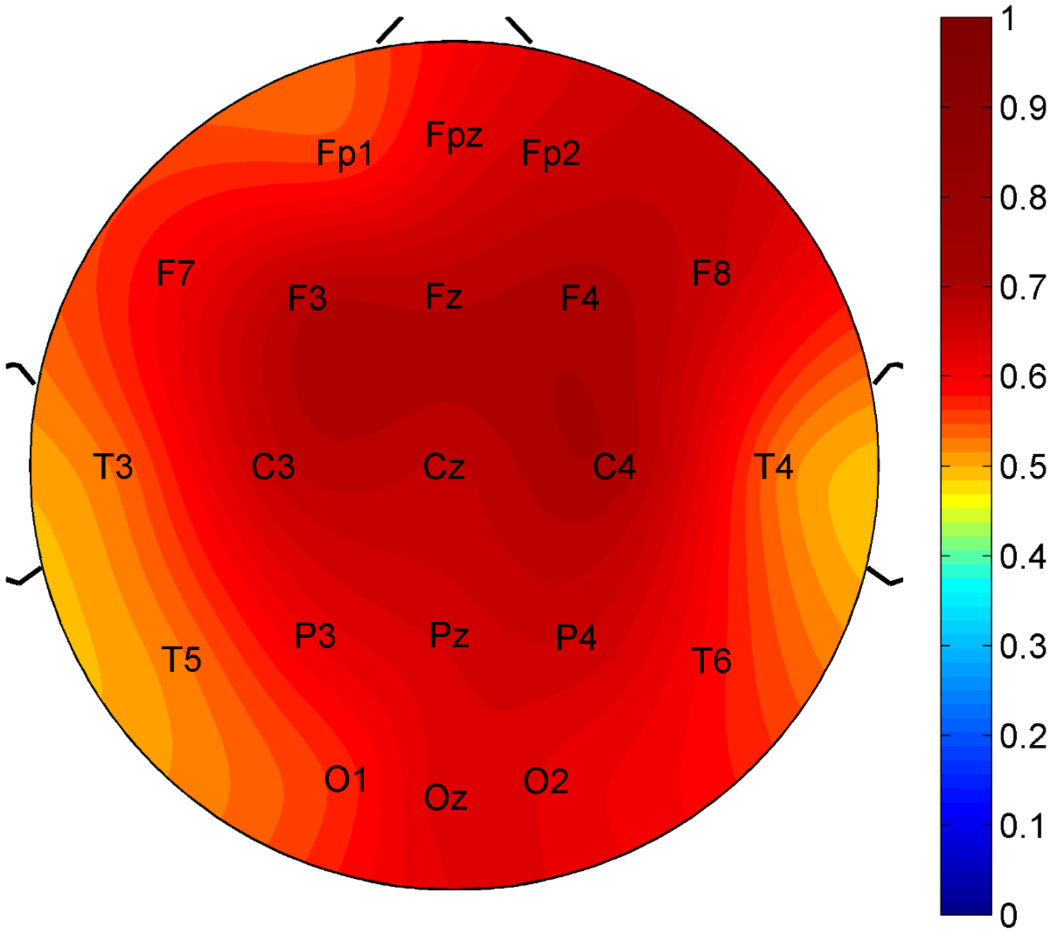

Supplementary Figure S1. Topographic plot of correlations of offline improvement in accuracy and 12.25 Hz z-scored EEG spectrum of NREM sleep in WS. Significance reached at r=0.55. Although the topographic plot of correlations indicate a spatially unspecific correlation between offline improvement in accuracy and 12.25 Hz z-scored EEG spectrum of NREM sleep in WS two points might be raised here. The first is that the maximum of the correlations closely corresponds with the functional localizational studies measuring motor cortex activation[1]. These regions were shown to be at the heart of the neural underpinnings of the FT improvement[2]. Thus, although the area of correlations is much more extended, the maximum fits the theoretical region of interest. The second point is that classical EEG localization is known to result in blurred images, extending well over the neural sources[3]. Thus, our findings might indicate a local, motor cortical effect which is spatially blurred according to the resolutional properties of the EEG. Another possible explanation is that not local but global spindles are responsible for the observed effects[4]. Further studies based on high density EEG or on other functional neuroimaging methods with superior localizational properties are needed in order to more precisely localize this effect.

References

1 Ball, T., Schreiber, A., Feige, B., Wagner, M., Lücking, C.H., & Kristeva-Feige, R. The Role of Higher-Order Motor Areas in Voluntary Movement as Revealed by High-Resolution EEG and fMRI. *NeuroImage*, **10** (6), 682–694. http://dx.doi.org/10.1006/nimg.1999.0507 (1999).

2 Karni, A., Meyer, G., Rey-Hipolito, C., Jezzard, P., Adams, M.M., Turner, R. & Ungerleider, L.G. The acquisition of skilled motor performance: Fast and slow experience-driven changes in primary motor cortex. *Proc. Natl. Acad. Sci. U S A* **95** 861-868, (1998).

3 Burle, B., Spieser, L., Roger, C., Casini, L., Hasbroucq, T., & Vidal, F. Spatial and temporal resolutions of EEG: Is it really black and white? A scalp current density view. International *Journal of Psychophysiology*, **97**(3), 210–220. http://doi.org/10.1016/j.ijpsycho.2015.05.004 (2015).

4 Piantoni, G., Halgren, E., & Cash., S.S. Spatiotemporal characteristics of sleep spindles depend on cortical location. *NeuroImage* **146**, 236-24. http://dx.doi.org/10.1016/j.neuroimage.2016.11.010. (2017)
